# Apolipoprotein B is regulated by gonadotropins and constitutes a predictive biomarker of IVF outcomes

**DOI:** 10.1186/s12958-016-0150-4

**Published:** 2016-05-21

**Authors:** Elodie Scalici, Shaliha Bechoua, Karine Astruc, Laurence Duvillard, Thomas Gautier, Véronique Drouineaud, Clément Jimenez, Samir Hamamah

**Affiliations:** ART-PGD Department, INSERM U1203, Arnaud de Villeneuve Hospital, CHU Montpellier, Montpellier, France; University of Burgundy, UFR Sciences de Santé de Dijon, Dijon, France; Department of Epidemiology, Bocage Hospital, CHU Dijon, Dijon, France; Biochemistry Department, CHU Dijon, Dijon, France; INSERM UMR 866, lipids, Nutrition and Cancer, UFR of Medicine, Dijon, France; ART Department, CHU de Dijon, Dijon, France; Service de Biologie de la Reproduction-CECOS, CHU de Bordeaux, Centre Aliénor d’Aquitaine, Bordeaux, France; ART-PGD Department, INSERM U1203, Arnaud de Villeneuve Hospital, CHU Montpellier, Montpellier, France

**Keywords:** Apolipoprotein B, Human follicular fluid, IVF outcomes, Human granulosa cells, Gonadotropins

## Abstract

**Background:**

Follicular fluid (FF) is an important micro-environment influencing oocyte growth, its development competence, and embryo viability. The FF content analysis allows to identify new relevant biomarkers, which could be predictive of in vitro fertilization (IVF) outcomes. Inside ovarian follicle, the amount of FF components from granulosa cells (GC) secretion, could be regulated by gonadotropins, which play a major role in follicle development.

**Methods:**

This prospective study included 61 female undergoing IVF or Intra-cytoplasmic sperm injection (ICSI) procedure. Apolipoprotein B (APOB) concentrations in follicular fluid and APOB gene and protein expression in granulosa cells from reproductively aged women undergoing an in vitro fertilization program were measured. The statistical analyses were performed according to a quartile model based on the amount of APOB level found in FF.

**Results:**

Amounts of APOB were detected in human FF samples (mean ± SD: 244.6 ± 185.9 ng/ml). The odds of obtaining an oocyte in the follicle and a fertilized oocyte increased significantly when APOB level in FF was higher than 112 ng/ml [i.e., including in Quartile Q 2, Q3 and Q4] (*p* = 0.001; *p* < 0.001, respectively). The probabilities of obtaining an embryo and a top quality embryo on day 2, were significantly higher if APOB levels were within the ranges of 112 and 330 ng/ml (i.e. in Q2 and Q3) or 112 and 230 ng/ml (i.e. in Q2), respectively (*p* < 0.001; *p* = 0.047, respectively). In addition, our experiments in vitro indicated that APOB gene and protein expression, along with APOB content into culture were significantly under-expressed in GC upon stimulation with gonadotropins (follicular stimulating hormone: FSH and/or human chorionic gonadotropin: hCG).

**Conclusion:**

We are reporting a positive and statistically significant associations between APOB and oocyte retrieval, oocyte fertilization, and embryo quality. Using an experimental study component, the authors report significant reduced APOB expression and content for luteinized granulosa cells cultured in the presence of gonadotropins.

**Electronic supplementary material:**

The online version of this article (doi:10.1186/s12958-016-0150-4) contains supplementary material, which is available to authorized users.

## Background

Apolipoprotein B (APOB) is a major structural protein for atherogenic APOB-containing lipoproteins, including chylomicrons, Very-Low-Density Lipoprotein (VLDL), Intermediate-Density Lipoprotein (IDL) and Low-Density Lipoprotein (LDL). The APOB-containing lipoproteins contain large amounts of cholesterol and triglycerides (TG) and are involved in lipoprotein metabolism and plasma lipid transport [1–4].

These lipoproteins are secreted by enterocytes [5], hepatocytes [6], human placenta [7] and cardiomyocytes [8]. APOB synthesis and secretion are regulated by several hormonal factors in hepatocytes such as Growth Hormone [9], insulin [10], adrenocorticotrophic hormone [11] and thyroid hormones [12]. Interestingly, we demonstrated previously that human granulosa cells (GC) are also able to assemble and secrete APOB100-containing lipoproteins [13]. Although GC functions are mostly influenced by gonadotropins, hormonal regulation of APOB expression in GC has never been investigated. Follicular fluid (FF), surrounding oocyte, provides an important microenvironment, influencing oocyte developmental competence and embryo development [14–16]. Therefore, recent works focused on FF analysis and identified in this fluid, potential biomarkers, which could be used to assess oocyte/embryo quality in order to improve the in vitro fertilization (IVF) success rates [17, 18]. Only few studies reported the presence of VLDL, LDL and APOB in human FF [13, 19, 20] and thus their relationships with IVF outcomes remain unknown.

This prospective translational study included two main objectives. First, we performed APOB quantification in individual FF samples (*n* = 201) from 61 patients undergoing IVF/Intra-cytoplasmic sperm injection (ICSI) program in order to investigate relationships between intra-follicular APOB levels and IVF/ICSI outcomes. Secondly, we explored hormonal regulation by gonadotropins of APOB expression in cultured human GC. To do so, in vitro studies were undertaken for which human GC isolated from FF were stimulated or not with gonadotropins (FSH and/or hCG). The apolipoprotein B gene and protein expressions in GC along with APOB content of the culture medium were investigated.

## Methods

### Determination of APOB concentrations in blood-free FF samples, obtained from 61 female patients

#### Patients' characteristics

A total of 61 female patients between 24 and 42 years of age (mean ± SD: 33.6 ± 4.8 years) and with a BMI between 16 and 35 kg/m^2^ (mean ± SD: 22.5 ± 3.8 kg/m^2^) were included in our prospective study. Among these patients, 16 were overweight or obese (BMI >25 kg/m^2^), whereas 3 were underweight (BMI < 18.5 kg/m^2).^ These women underwent controlled ovarian stimulation (COS) followed by either IVF (*n* = 34) or ICSI (*n* = 27) procedure at the Assisted Reproduction Technology Department of the Dijon hospital (CHU de Dijon, France). Exclusion criteria included polycystic ovary syndrome or lipid metabolic disorders [21]. On average, the delay in conception was 3.6 ± 1.8 years. Forty three couples had primary infertility (70.5 %) and 18 couples secondary infertility (29.5 %). Fifty five couples were enrolled in IVF/ICSI procedure for the first or second time. Female infertility, sperm abnormalities, mixed and unexplained infertility were diagnosed in 49.2 %, 37.7 %, 8.2 %, and 4.9 % of the cases, respectively. Based on anti-müllerian hormone (AMH) and antral follicular count (AFC), all women had a normal ovarian reserve. The patients' clinical characteristics are summarized in Additional file 1: Table S1. Informed consents were obtained from all patients for the use of FF samples on the day of oocyte retrieval.

#### IVF/ICSI procedure

Sixty one patients underwent an agonist GnRH protocol including ovarian stimulation with recombinant FSH (r-FSH) (Puregon, Schering Plough, Courbevoie, France or Gonalf, Merck Serono, Lyon, France). Ovarian stimulation response was monitored by both, serum estradiol concentration and transvaginal ultrasound assessment of follicular and endometrial growth.

Ovulation was induced with 6500 UI of recombinant hCG (Ovitrelle, Merck Serono, Lyon, France) when at least three follicles had reached 17 mm or more in diameter. Cumulus oocyte complexes (COC) were retrieved by transvaginal ultrasound-guided aspiration, 36 h after ovulation triggering, rinsed twice and isolated for conventional IVF or ICSI protocol. In ICSI procedure, after denudation, oocyte maturity was assessed and mature oocytes were micro-injected. Oocytes were individually maintained in 30 μl microdrops of culture medium (Global Medium, LifeGlobal, USA) under oil at 37 °C in 6 % CO_2_ and 5 % O_2_. Normal fertilization was checked between 16 and 18 h after insemination or microinjection by the presence of two pronuclei (2PN) and the two polar bodies. Early cleavage (EC) was evaluated 25 or 27 h after microinjection (ICSI) or insemination (IVF), respectively. Two days after oocyte retrieval, embryo quality was scored according to morphological criteria: (i) cleavage stage, (ii) number and size of blastomeres, and (iii) degree of fragmentation. An embryo was considered as a top quality embryo if there were 4 to 5 blastomeres on day 2, less than 20 % of fragments, and no multinucleation [22]. On day 2, the embryo(s) (standard-of-care in the centre) was (were) transferred under transabdominal ultrasound guidance.

#### APOB quantification by ELISA in individual FF samples

For the 61 patients, at the day of oocyte retrieval, all the follicles visualized by ultrasound were aspirated individually without flushing. Only blood-free FF samples (*n* = 201) were collected and centrifuged at 3000 g for 15 min. The supernatants were removed and stored at –80 °C for APOB quantification. APOB concentration in each FF sample was quantified using the Human APOB ELISA^pro^kit (no. 3715-1HP-2 Mabtech AB, Sophia Antipolis, France). Inter and intra-assay variation coefficients were 10.0 % (CV) and 2.0 % (CV), respectively.

#### Quartile model

Quartiles (Q) of APOB concentrations (ng/ml) measured in the FF (*n* = 201) samples of the 61 patients were defined as followed:

- Q1: APOB <112 ng/ml
- Q2: 112 <APOB<230 ng/ml
- Q3: 230 <APOB<330 ng/ml
- Q4: APOB>330 ng/ml

The patients’ characteristics in each defined quartile are presented in Additional file 2: Table S2.

See statistical analysis section for further information.

### Are apolipoprotein B gene expression and protein level regulated in vitro by gonadotropins?

#### CG isolation

GC were isolated from FF samples, collected the day of oocyte retrieval, as previously described [23]. Typically, FF from 6 or 7 women (undergoing oocyte retrieval the same day) with normal ovarian reserve were pooled and centrifuged at 300 g for 5 min and at 500 g for 5 min, successively. This experiment was performed 10 times (*n* = 10). The cell layer was removed and re-suspended in DMEM/F12 medium supplemented with 10 % foetal calf serum (FCS) and antibiotics (100 UI/ml of penicillin and 100 μg/ml streptomycin) (Invitrogen, France). Then, to separate GC from cellular debris, the cell suspension was centrifuged using a Puresperm gradient (Nidacon, Mölndal, Sweden). The GC layer was collected, washed and re-suspended in DMEM/F12 medium. In order to eliminate macrophages, GC suspension was allowed to settle for 20 min and the supernatant was centrifuged at 400 g for 30 min. The final pellet was re-suspended in 1 ml of DMEM/F12 medium and the cells suspension was distributed in each well at a density of 2 × 10^5^ cells.

#### Gonadotropin stimulation of GC primary culture

GC were cultured in DMEM/F12 medium supplemented with 10 % FCS and antibiotics for 72 h to regain a more native reaction pattern before hormonal stimulation [24]. After three days in culture, GC were either untreated (control) or treated for 48 h with 30 ng/ml of r-FSH (Puregon®, Schering Plough, Courbevoie, France) or 30 ng/ml of hCG (Ovitrelle ®, Merck Serono, Lyon, France), individually or in combination. One μl of Androstenedione (Sigma, Poole, UK) was added to the culture medium to provide specific substrate to GC, for oestrogen synthesis. Therefore, 17 β-estradiol quantification by immuno-chemiluminescence using a commercially kit (ADVIA® Centaur, Bayer Diagnostics, Tarrytown, NY, USA) allowed to check GC viability (Additional file 3: Figure S1). From day 3, FCS was replaced in culture medium by Nutridoma-CS (Roche Applied Science, Germany) in order to avoid lipoproteins contamination. After 5 days of culture, culture medium and GC samples were retrieved and stored at –80 °C. Each culture’s experiment was performed in triplicate and 10 times.

#### Quantitative (real-time) PCR: APOB gene expression in GC

Total RNA from GC was extracted using the Absolutely RNA Microprep kit (ref 400805, Agilent Technologies, Jolla, USA) according to manufacturer’s recommendations and quantified by Nanodrop ND-1000. By adding 10 μl of RT Buffer and 1 μl of RT Enzyme Mix, 200 ng of total RNA was reverse-transcribed into cDNA using the High Capacity RNA-to cDNA kit (Applied Biosystems, Foster City, USA). Quantitative PCR was performed from 5 μl of cDNA, 10 μl of Master Mix, 1 μl of Master Assay and 4 μl of H_2_O, by using Taqman technology (Applied Biosystems, Foster City, USA). Each qPCR reaction contained specific primers of *APOB* gene (ref 4331182, ID assay: Hs00181142_m1; Applied Biosystems). The human housekeeping *GAPDH* gene (ref 4351372, ID assay: Hs04420697_g1; Applied Biosystems) was added in each qPCR template, as an endogenous control to normalize relative *APOB* gene expression in untreated (control) and treated GC samples. The *GAPDH* housekeeping gene was stably expressed in GC pools (*n* = 10) from different FF samples (Additional file 4: Figure S2). The *APOB* relative expression was calculated according to the equation 2^−*ΔΔ*CT^method.

#### Western blot analysis: APOB protein expression in GC

After the addition of LDS Sample buffer and reducing agent (Nupage, Invitrogen, France) to GC samples and incubation at 95 °C for 10 min, each sample (containing 30 μg of total protein per well) was applied onto 4-12 % polyacrylamide gradient gels (NuPage, Invitrogen, France) inserted in a X-Cell SureLock system (Invitrogen, France) and then blotted to nitrocellulose membranes (Protan, Schleicher and Schuell, Dassel, Germany). The membrane was saturated during one hour with Phosphate Buffered Saline (PBS), 0.1 % Tween (PBS-T 0.1 %) and 5 % low-fat dried milk. An incubation was performed overnight at 4 °C, with a rabbit polyclonal human anti-ApoB100 antibody (H-300) (1:1000, sc- 25542, Biotechnology, Santa Cruz, USA), in PBS-T 0.1 %. Then, the membrane was incubated for one hour with an anti-rabbit HRP antibody (Biotechnology, Santa Cruz, USA) diluted in PBS-T 0.1 % (1:5000). APOB was detected using Supersignal West Pico Trial kit (Thermoscientific, Rockford, USA) and ChemiDocTM XRS Embellish-Images LabTM 2.0 Software system (Bio-Rad).

#### Slot Blot analysis: APOB content of the culture medium

Culture medium samples (300 μl) were applied onto nitrocellulose membrane (Protan, Schleicher and Schuell, Dassel, Germany) and the membrane was washed with Tris Buffer Saline (TBS) with 0.1 % of Tween-20 (TBS-T 0.1 %), then saturated in TBS-T 0.1 % containing 5 % low-fat dried milk for one hour. The incubations were performed as described for western blots. Slot blots were detected using the Supersignal West Pico Trial kit (Thermoscientific, Rockford, USA) and analyzed using the ChemiDocTM XRS Embellish-Images LabTM 2.0 Software system (Bio-Rad).

#### Statistical analyses

The means ± standard deviations (SD) and 95 % confidence intervals (CI) were reported for quantitative data and percentages for categorical ones. The comparisons of APOB concentrations according to IVF/ICSI outcomes were performed using Student’s *t*-test. Then, simple linear regression models with robust variance estimates for cluster-correlated data were performed to analyze relationships between patients’ characteristics and APOB concentration in FF. FF produced by the same women defined the cluster. FF APOB concentration effects on IVF/ICSI outcomes were estimated using univariate logistic regression. For each parameter, we compared the model including APOB concentration as a continuous variable with the model including APOB concentration quartiles as qualitative variables, to select and report the best one. The first quartile (Q1) was taken as the reference for the comparison with the other quartiles. Then, prognostic effect of patient’s characteristics on IVF/ICSI outcomes was estimated using univariate logistic regression models. Each variable with *p* < 0.2 was included in the multivariate logistic regression models and used to estimate the independent effect of APOB concentration in FF on IVF/ICSI outcomes. For all these logistic regression models, robust variance estimations for cluster-correlated data were used.

For in vitro studies, Mann-Whitney tests were used to compare *APOB* gene expression in treated and untreated GC.

Statistical analyses were performed using Stata 10.0 software. Results were considered significant when *p*-value < 0.05.

#### Ethical approval

Ethical approval was obtained from the local Institutional Review Board**,** CHU of Dijon, France.

## Results

### APOB is present in human follicular fluid and provides a new biomarker to predict oocyte and embryo quality

#### Relationships between APOB concentrations in FF and patient’s characteristics (age, BMI, number of attempts)

Follicular fluid from young patients (<36 years, *n* = 41) contained significant higher APOB levels than FF obtained from older patients (>36 years, *n* = 20) (*β* = –93.6, *p* = 0.02). Likewise, APOB levels in FF were significantly higher for patients with normal BMI compared to underweight (BMI < 18.5 kg/m^2^) or obese patients (BMI > 30 kg/m^2^) (*β* = –162.2, *p* = 0.001 and *β* = –142.3, *p* = 0.005, respectively). In addition, FF related to women enrolled in a first or second attempt contained higher APOB levels than patients undergoing their third or fourth attempt (*β* = –148.8, *p* = 0.005) (Table 1).

**Table 1:** Relationships between APOB concentrations in human FF and clinical prognostic markers of IVF success

Variable	β	Robust SE	*p-value*
Age (years)			
Age >36 *versus* ≤ 36	–93.6	37.7	0.02
BMI (kg/m^2^) 18.5 ≤ BMI < 25 (reference)	−	−	−
BMI < 18.5	–162.2	48.0	0.001
25 ≤ BMI < 30	–59.0	72.7	NS
BMI ≥ 30	–142.3	48.6	0.005
Number of attempts			
3 and 4 *versus* 1 and 2	–148.8	51.3	0.005

*BMI* Body Mass Index, *β* regression coefficient, *SE* Standard Error, *NS* No significant

#### APOB concentrations in individual FF samples according to IVF/ICSI outcomes

Amounts of APOB were detected in 201 individual FF samples from pre-ovulatory follicles (mean ± SD: 244.6 ± 185.9 ng/ml, median: 229.3 ng/ml). In 90 % of the samples analyzed (*n* = 182), APOB concentration was under 480 ng/ml. Table 2 shows that APOB concentration was significantly higher in FF samples containing an oocyte (*n* = 143) than those related to empty zona pellucida (*n* = 58) (271 ± 181.8 ng/ml *versus* 179 ± 181.3 ng/ml, *p* = 0.001, respectively). Likewise, FF samples related to normal fertilized oocytes (*n* = 107, fertilization rate = 82.3 %, IC95 [75.7–89]) contained significant increased APOB levels compared to those related to no fertilized oocytes (*n* = 23) (279.8 ± 168.0 ng/ml *versus* 162.6 ± 192.5 ng/ml, *p* = 0.004, respectively). APOB levels were significantly also increased in FF samples associated with an embryo on day 2 (*n* = 92) compared to those associated with no embryo (*n* = 109) (294.3 ± 159.8 ng/ml *versus* 202.6 ± 196.5 ng/ml, *p* < 0.001, respectively).

**Table 2:** Comparisons of APOB concentrations in human FF according to IVF/ICSI outcomes

FF related to	n / number of FF analyzed (%)	APOB (ng/ml)		*p-value*
		Mean ± SD	[95 % CI]	
Oocyte	143 / 201 (71.1)	271.0 ± 181.8	[240.9; 301.0]	*p* = 0.001
Empty zona pellucida (no oocyte)	58 / 201 (28.9)	179.0 ± 181.3	[131.8; 227.2]	
Normal fertilized oocytes	107 / 201 (53.2)	279.8 ± 168	[247.6; 312.0]	*p* = 0.004
No fertilized oocytes	23 / 201 (11.4)	162.6 ± 192.5	[79.4; 245.8]	
Embryo	92 / 201 (45.8)	294.3 ± 159.8	[261.2; 327.4]	*p* < 0.001
No embryo	109 / 201(54.2)	202.6 ± 196.5	[165.3; 239.9]	
Early cleavage	22 / 92 (23.9)	352.1 ± 192.6	[266.7; 437.5]	NS
No early cleavage	70 / 92 (76.1)	276.1 ± 144.9	[241.6; 310.7]	
Top quality embryo	45 / 92 (48.9)	322.1 ± 205.2	[260.4; 383.7]	NS
No top quality embryo	47 / 92 (51.1)	267.7 ± 93.6	[240.2; 295.2]	

Values are reported by the number of variable (n) / number of FF analyzed (percentage of the total). The comparisons were performed using Student’s *t*-test. *SD* Standard Deviation, *FF* Follicular Fluid, *NS* No significant

Moreover, when considering APOB divided into quartiles (Table 3), we found significant associations between APOB concentrations in FF and the probabilities to obtain an oocyte, a fertilized oocyte, an embryo and a top quality embryo on day 2 (*P* = 0.001; *P* < 0.001, *P* < 0.001, *P* = 0,047, respectively). Indeed if APOB levels were equal or higher to 112 ng/ml (i.e. including in Q2, Q3 or Q4), the odds of obtaining an oocyte, a normal fertilized oocyte, an embryo or a top quality embryo were significantly increased (Conditional Odds Ratio, COR: 3.9, 4.2 and 3.9; COR: 6.2, 10.1 and 6.4; COR: 15.2, 15.5 and 8; COR: 4.4, 4.2, 4.4 in Q2, Q3 and Q4, respectively). Moreover, after adjustment for patients’ characteristics, if APOB concentrations were above 112 ng/ml and within the ranges of Q2, Q3 or Q4, the probabilities of obtaining an oocyte and a fertilized oocyte remained significantly higher (AOR: 3.4, 3.3 and 3.4; AOR: 7.3, 11.2 and 6.3 in Q2, Q3 and Q4, respectively). However, after adjustment for patient’s characteristics, the odds of obtaining an embryo remained significantly elevated only if APOB concentrations were included in Q2 and Q3 (between 112 and 330 ng/ml) (AOR: 17.3, 15.5 in Q2 and Q3, respectively). Finally, the concentrations of APOB that could predict the odds of obtaining a top quality embryo on day 2 (AOR = 4.8) were comprised between 112 and 230 ng/ml (i.e. including in Q2).

**Table 3:** Probabilities of APOB concentration in human FF, divided into quartiles (Q), to predict IVF/ICSI outcomes

APOB quartiles (ng/ml)	n / number of FF analyzed (%)	*P*	Crude OR [95 % CI]	*p-value*	Adjusted OR [95 % CI]^a^	*p-value*
Probabilities of obtaining an oocyte						
Q1 (<112)	24/50 (48.0)	0.001	-- (reference)		-- (reference)	
Q2 (≥112, <230)	40/51 (78.4)		3.9 [1.65–9.4]	0.002	3.4 [1.04–11.2]	0.044
Q3 (≥230, <330)	39/49 (79.6)		4.2 [1.73–10,3]	0.002	3.3 [1.19–9.08]	0.022
Q4 (≥330)	40/51 (78.4)		3.9 [1.65–9.4]	0.002	3.4 [1.01–11.44]	0.049
Probabilities of obtaining a fertilized oocyte						
Q1(<112)	12/50 (24.0)	<0.001	-- (reference)		-- (reference)	
Q2 (≥112, <230)	34/51 (66.7)		6.2 [1.79–21.76]	0.004	7.3 [1.59;33.43]	0.011
Q3 (≥230, <330)	33/49 (67.3)		10.1 [2.38–42.69]	0.002	11.2 [1.67;75.63]	0.013
Q4 (≥330)	28/51 (55.0)		6.4 [1.69–24.37]	0.006	6.3 [1.13;34.86]	0.035
Probabilities of obtaining an embryo on day 2						
Q1(<112)	5/50 (10.0)	<0.001	-- (reference)		-- (reference)	
Q2 (≥112, <230)	32/51 (62.8)		15.2 [5.11–44.95]	<0.001	17.3 [2.2;137.1]	0.007
Q3 (≥230, <330)	31/49 (63.3)		15.5 [5.19–46.29]	<0.001	15.5 [1.9;130.3]	0.011
Q4 (≥330)	24/51 (47.1)		8 [2.72–23.51]	<0.001	8.3 [0.8;82.0]	0.071
Probabilities of obtaining a top quality embryo on day 2						
Q1(<112)	4/50 (8.0)	0.047	-- (reference)		-- (reference)	
Q2 (≥112, <230)	14/51 (27.5)		4.4 [1.32–14.38]	0.016	4.8 [1.04;22.41]	0.045
Q3 (≥230, <330)	13/49 (26.5)		4.2 [1.24–13.86]	0.021	4.1 [0.75;22.64]	NS
Q4 (≥330)	14/51 (27.4)		4.4 [1.32–14.38]	0.016	3.9 [0.64;23.91]	NS

^a^Probabilities to obtain an oocyte and an embryo were adjusted for age and aetiology of infertility. Probabilities to obtain a fertilized oocyte and a top embryo were adjusted for age. Q1, quartile 1; Q2, quartile 2; Q3, quartile 3; Q4, quartile 4. Q1 was considered as the reference. *OR* Odds Ratio, *CI* confidence intervals, *FF* Follicular Fluid, *NS* No significant

### Regulation in vitro of apolipoprotein B gene and protein by gonadotropins

#### APOB gene expression in GC stimulated by gonadotropins

**Fig. 1:**
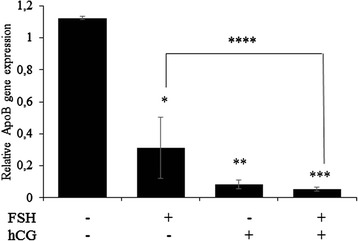
*APOB* gene expression profile in human granulosa cells treated with FSH, hCG individually or in combination, compared to untreated cells (control) (*p < 0.001, **p < 0.001, ***p < 0.001, ****p = 0.019, respectively). The relative *APOB* gene expressions (±SD) were reported for each culture’s condition performed in triplicates and 10 times

*APOB* gene was significantly under-expressed in treated human GC compared to untreated cells (control). Indeed *APOB* gene expression decreased significantly after a treatment by FSH, hCG or FSH and hCG compared to control (*p* < 0.001; *p* < 0.001; *p* < 0.001, respectively). Moreover, the down-regulation of *APOB* gene expression by FSH was significantly strengthened after introduction of hCG into the culture medium (FSH versus FSH and hCG, *p* = 0.019) (Fig. 1).

#### APOB protein expression in GC stimulated by gonadotropins

**Fig. 2:**
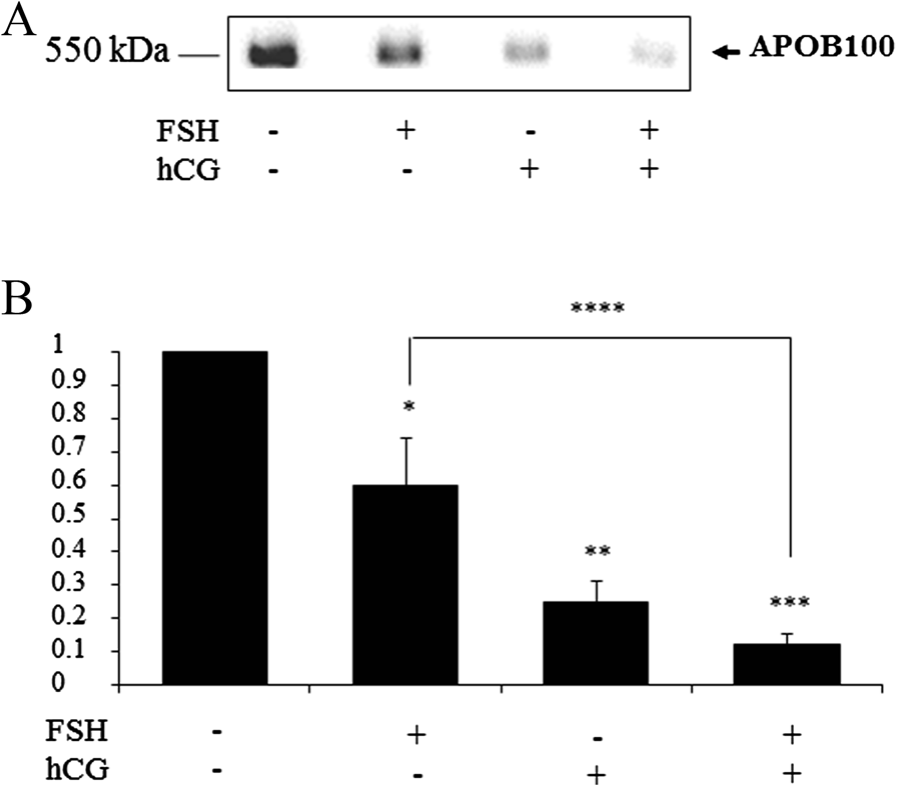
**a**. Representative Western blot of APOB expression in human granulosa cells treated with FSH, hCG, individually and in combination compared to untreated cells (control); **b**. The APOB-100 band intensities were compared between GC stimulated by FSH, hCG or FSH and hCG and unstimulated GC (control) (*p = 0.045; **p = 0.009; ***p = 0.005, respectively). FSH versus FSH and hCG, ****p = 0.01

As shown in Fig. 2a, an APOB band (APOB100) was detected in human GC samples. The intensity of this band was significantly decreased in GC stimulated by FSH, hCG or FSH and hCG compared to unstimulated GC (control) (*p* = 0.045; *p* = 0.009; *p* = 0.005, respectively) (Fig. 2a and 2b). Moreover, APOB protein expression was significantly lower in GC, treated simultaneously with FSH and hCG compared to those treated only by FSH (FSH versus FSH and hCG, *p* = 0.01) (Fig. 2b).

#### APOB protein content in the culture medium

**Fig. 3:**
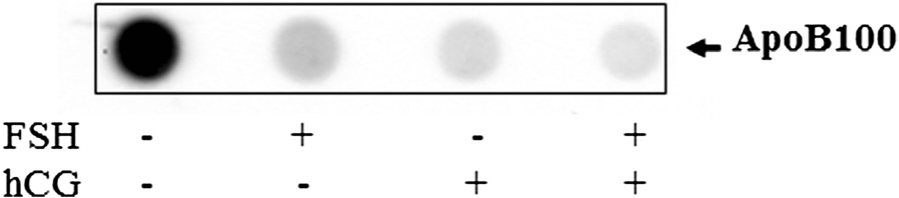
Representative slot blots of APOB content into culture medium from granulosa cells treated with FSH, hCG, individually and in combination compared to untreated cells (control)

Likewise, APOB was identified into culture medium (Fig. 3). Low APOB secreted into culture medium was found when GC were stimulated with gonadotropins, compared to control (no treatment). The inhibitory effect was synergized when the stimulation of GC was performed with both, FSH and hCG (Fig. 3).

## Discussion

This study investigated the relationships between APOB concentrations in individual FF samples and IVF/ICSI outcomes, and the hormonal regulation of APOB expression in human cultured GC. Our results suggest that APOB could be used as a non-invasive biomarker to predict oocyte and embryo outcomes in patients undergoing IVF/ICSI procedure. Moreover, APOB is down-regulated by gonadotropins (FSH and hCG) which could influence APOB content in ovarian follicle during COS. Several clinical parameters influence IVF/ICSI prognostic and outcomes. Interestingly, we showed that APOB concentration in FF was significantly related to some clinical characteristics. Indeed, APOB level appeared to be significantly higher in FF from women with good prognostic clinical markers such as young age, normal BMI and first or second IVF attempt. Aging is known to be associated with alterations of follicular environment and follicular cell functions, resulting in a decrease of some metabolic components present in the FF [25]. Accordingly, intra-follicular APOB content could decrease with advanced age, leading to harmful effects on oocyte and embryo development. By contrast, one study reported low APOB levels in FF from young women (<35 years) compared to older women (>40 years) [20]. However, these results included a very low number of patients (*n* = 5 for young women; *n* = 4 for old women) compared to our study (*n* = 61). Likewise, increasing BMI was also associated with a decrease of APOB concentrations in FF which could be related to the follicular environment impairment in obese women at risk for poor reproductive performance [26]. One study investigated FF composition according to BMI classification. However in this study, APOB was not detected in the FF samples, most probably because of a low-sensitivity of the APOB assay method used [27]. In addition, it was reported that pregnancy rates decreased with repeated IVF attempts [28]. Therefore, women enrolled in a first or second attempt, have clinical parameters for prediction of success, related to a good health of recruited ovarian follicles which contain higher APOB concentrations. Moreover, the use of repeated ovarian stimulation treatment could induce changes in the composition of the follicular fluid [29]. This could explain the significant variations of intra-follicular APOB content according to the number of IVF attempts. Some FF components are involved in oocyte growth, oocyte developmental competence and embryo development. They provide non-invasive biomarkers of oocyte and/or embryo quality [17, 18]. Likewise, we demonstrated that APOB concentration in FF was significantly associated with the probability to obtain an oocyte in the follicle, a normal fertilization, an embryo and a top quality embryo, respectively. Using a quartile model, we were able to demonstrate that the intra-follicular APOB level could be used to predict significantly and independently oocyte and embryo outcomes. It is conceivable that APOB in FF could play a role in the lipid transport as in plasma [1] and could supplies cholesterol and triglycerides (TG) to oocytes for supporting early embryo development. Indeed, TG act as an energy source during oocyte maturation and potentially during pre-implantation embryo development [30, 31]. Moreover, the pre-implantation embryo needs amounts of cholesterol for membrane formation during its cell cleavages [32–37]. Interestingly, LDL and VLDL receptors were already identified on chicken and mouse oocytes [38–40], but their presence have never been investigated in humans. In addition, a very high intra-follicular APOB level could affect embryo development and viability. Indeed, it is noteworthy to mention that an APOB concentration within the range of 112 and 230 ng/ml in FF appeared to be the optimal dose to obtain a top quality embryo. A large amount of APOB could reflect a lipid-rich environment that could have a negative impact on oocyte quality and thus embryo development [41]. In addition, our data suggest that APOB could constitute a new promising biomarker, offering an additional tool to select the best embryo for replacement in order to improve IVF success rates.

Moreover, we reported in a previous work that GC have the ability to assemble and secrete ApoB100-containing lipoproteins [13]. Therefore, the second part of this present study consisted in understanding how the synthesis of APOB by GC could be regulated by gonadotropins and this knowing that these hormones are widely used in the field of IVF when performing ovarian stimulation. We showed that stimulation of cultured GC by gonadotropins (FSH or/and hCG) modified APOB expression and led to a significant decrease of its expression at the gene and protein level. In addition, GC treatment by gonadotropins reduced APOB protein content into the culture medium. Here, we are for the first time demonstrating that gonadotropins can regulate APOB level in an in vitro GC model. During IVF procedure, COS could contribute to modify FF composition and thus GC secretions [29]. One of the hypothesis would be that APOB is under-expressed in human GC upon a strong stimulation with gonadotropins. This, would create a poor availability of APOB in the follicular micro-environment leading to a disruption of lipid transfer from GC to oocyte.

One can reasonable assume that a young woman with good prognostic factors (normal BMI and first or second attempt) will need less FSH doses during ovarian stimulation than a woman with compromised prognostic factors (older woman, overweight). Indeed, this has been confirmed in our prospective study where we found that the “good prognosis” women needed lower dose of r-FSH than the “bad prognosis” women (1892.4 ± 756.6 IU versus 2461 ± 932.46 IU, *p* = 0.006, respectively, data not shown). Therefore, the results obtained from the experiments in vitro (down regulation of APOB by FSH) could explain why APOB level was found significantly lower in patients with compromised prognostic factors compared to patients with good prognostic factors.

## Conclusions

Our findings suggest that APOB could be easily quantified in order to identify and select among the embryos, the one that display all the morphological characteristics needed for a pregnancy to occur. Hence, APOB could be used as a prognostic biomarker when performing in vitro fertilization. Moreover, we are reporting for the first time a new regulation of APOB by gonadotropins in an in vitro human GC model. APOB was found to be down-regulated by gonadotropin treatment in cultured human GC, suggesting a potential effect of COS on intra-follicular fluctuation of APOB concentrations during IVF/ICSI procedure.

## Additional files

Additional file 1: Table S1. Patients' characteristics. All values are reported by the mean ± SD (Standard Deviation) or the number of subjects (percentage of the total). BMI, Body Mass Index; IVF, In Vitro Fertilization; ICSI, Intracytoplasmic Sperm Injection. (DOCX 12 kb) Additional file 2: Table S2. Description of patients' characteristics according to APOB concentrations divided into quartiles. Q1, quartile 1; Q2, quartile 2; Q3, quartile 3; Q4, quartile 4; SD, Standard Deviation; CI, Confidence Intervals, BMI, Body Mass Index; IVF, In Vitro Fertilization; ICSI, Intracytoplasmic Sperm Injection. (DOCX 15 kb) Additional file 3: Figure S1. 17 β-estradiol quantification (ng/l) into culture medium by immuno-chemiluminescence. Androstenedione (And) was added to the culture medium to provide specific substrate to granulosa cells, for oestrogen synthesis. (TIF 69 kb) Additional file 4: Figure S2. The constant *GAPDH* gene expression in GC pools (*n* = 10) under our experiment conditions. All Ct-values were reported for the different GC pools from FF samples. (JPG 137 kb)

## Abbreviations

APOB: apoliprotein B
AFC: antral follicular count
AMH: anti-mullerian hormone
BMI: body mass index
CI: confidence intervals
COC: cumulus oocyte complexes
EC: early cleavage
FCS: fetal calf serum
FF: follicular fluid
FSH: follicular stimulating hormone
GC: granulosa cells
GnRH: gonadotropin releasing hormone
hCG: human chorionic gonadotropin
IVF: in vitro fretilization
ICSI: intracytoplasmic sperm injection
PCR: polymerase chain reaction
Q: quartile
SD: standard deviation
