# Supplementary material for: Apolipoprotein B is regulated by gonadotropins and constitutes a predictive biomarker of IVF outcomes
Source: Reprod Biol Endocrinol. 2016 May 21;14:28. doi: 10.1186/s12958-016-0150-4 (PMC4875704; doi:10.1186/s12958-016-0150-4)
Supplement: Additional file 1: Table S1. — Patients' characteristics. All values are reported by the mean ± SD (Standard Deviation) or the number of subjects (percentage of the total). BMI, Body Mass Index; IVF, In Vitro Fertilization; ICSI, Intracytoplasmic Sperm Injection. (DOCX 12 kb) [file 12958_2016_150_MOESM1_ESM.docx]

**Supplemental Table 1:**

| Variable | Total (n=61) |
| --- | --- |
| Age (years ± SD) | 33.6 ± 4.8 |
| age ≤ 36 (%) | 41 (67.2) |
| age > 36 (%) | 20 (32.8) |
| BMI (kg/m2 ± SD) | 22.5 ± 3.8 |
| 18,5≤BMI<25 (%) | 42 (68.9) |
| BMI<18,5 (%) | 3 (4.9) |
| 25≤BMI<30 (%) | 12 (19.7) |
| BMI≥30 (%) | 4 (6.5) |
| Infertility period (years ± SD) | 3.6 ± 1.8 |
| Primary infertility (%) | 43 (70.5) |
| Secondary infertility (%) | 18 (29.5) |
| Aetiologies of infertility | |
| Sperm abnormalities (%) | 23 (37.7) |
| Endometriosis (%) | 14 (23) |
| Tubal disease (%) | 12 (19.7) |
| Mixed infertility (%) | 5 (8.2) |
| Ovarian disorder (%) | 3 (4.9) |
| Unexplained infertility (%) | 3 (4.9) |
| Anovulation or dysovulation (%) | 1 (1.6) |
| Number of attempts | |
| 1 (%) | 46 (75.4) |
| 2 (%) | 9 (14.8) |
| 3 (%) | 2 (3.3) |
| 4 (%) | 4 (6.5) |
| IVF (%) | 34 (55.7) |
| ICSI (%) | 27 (44.3) |
