# Supplementary material for: Apolipoprotein B is regulated by gonadotropins and constitutes a predictive biomarker of IVF outcomes
Source: Reprod Biol Endocrinol. 2016 May 21;14:28. doi: 10.1186/s12958-016-0150-4 (PMC4875704; doi:10.1186/s12958-016-0150-4)
Supplement: Additional file 2: Table S2. — Description of patients' characteristics according to APOB concentrations divided into quartiles. Q1, quartile 1; Q2, quartile 2; Q3, quartile 3; Q4, quartile 4; SD, Standard Deviation; CI, Confidence Intervals, BMI, Body Mass Index; IVF, In Vitro Fertilization; ICSI, Intracytoplasmic Sperm Injection. (DOCX 15 kb) [file 12958_2016_150_MOESM2_ESM.docx]

**Supplemental Table 2:**

|  | APOB quartiles (ng/ml) | | | | |
| --- | --- | --- | --- | --- | --- |
| Variable  mean± SD  [95% CI] | Q1 (<112)  25.1 ± 30.3  [16.4; 33.7] | Q2 (≥112, <230)  186.3 ± 32.8  [177.1; 195.6] | Q3 (≥230, <330)  277.0 ± 30.4  [268.3; 285.7] | Q4 (≥330)  486.8 ± 151.1  [444.3; 529.3] | Total  224.6 ± 185.9  [218.7; 270.4] |
|  | n=50 | n=51 | n=49 | n=51 | n=201 |
| Age (years):  mean± SD [95%CI] | 33.6 ± 5.0  [32.1; 35.0] | 34.9 ± 4.1  [33.7; 36.1] | 33.8 ± 4.2  [32.6; 35.0] | 32.1 ± 3.7  [31.0; 33.1] | 33.6 ± 4.4  [33.0; 34.2] |
| age ≤ 36 (%) | 34 (68.0) | 31 (60.8) | 34 (69.4) | 47 (92.2) | 146 (72.6) |
| age > 36 (%) | 16 (32.0) | 20 (39.2) | 15 (30.6) | 4 (7.8) | 55 (27.4) |
| BMI (kg/m2):  mean± SD [95%CI] | 22.9 ± 4.7  [21.5 ; 24.3] | 21.9 ± 3.9  [20.7; 23.1] | 22.8 ± 3.5  [21.7; 23.9] | 22.1 ± 2.1  [21.5;22.8] | 22.4 ± 3.7  [21.9; 22.9] |
| 18,5≤BMI<25 (%) | 24 (48.0) | 41 (80.4) | 34 (69.4) | 43 (84.3) | 142 (70.6) |
| BMI<18,5 (%) | 7 (14.0) | 3 (5.9) | 0 (0) | 0 (0) | 10 (5.0) |
| 25≤BMI<30 (%) | 13 (26.0) | 2 (3.9) | 12 (24.5) | 8 (15.7) | 35 (17.4) |
| BMI≥30 (%) | 6 (12.0) | 5 (9.8) | 3 (6.1) | 0 (0) | 14 (7.0) |
| Infertility period:  mean ± SD [95%CI] | 4.6 ± 1.8  [4.1; 5.1] | 4.0 ± 1.4  [3.6; 4.4] | 3.7 ± 1.8  [3.2; 4.3] | 2.6 ± 1.1  [2.3; 2.9] | 3.7 ± 1.7  [3.5 ; 4.0] |
| Primary infertility (%) | 23 (46.0) | 33 (64.7) | 38 (77.6) | 44 (86.3) | 138 (68.7) |
| Secondary infertility(%) | 27 (54.0) | 18 (35.3) | 11 (22.4) | 7 (13.7) | 63 (31.3) |
| Aetiology of infertility |  |  |  |  |  |
| Sperm abnormalities (%) | 17 (34.0) | 15 (29.4) | 16 (32.6) | 24 (47.1) | 72 (35.8) |
| Endometriosis (%) | 11 (22.0) | 6 (11.8) | 10 (20.4) | 13 (25.5) | 40 (19.9) |
| Tubal disease (%) | 6 (12.0) | 14 (27.5) | 16 (32.7) | 10 (19.6) | 46 (22.9) |
| Mixed infertility (%) | 13 (26.0) | 5 (9.8) | 2 (4.1) | 2 (3.9) | 22 (10.9) |
| Ovarian disorder (%) | 1 (2.0) | 4 (7.8) | 3 (6.1) | 0 (0) | 8 (4.0) |
| Unexplained infertility (%) | 2 (4.0) | 5 (9.8) | 2 (4.1) | 2 (3.9) | 11 (5.5) |
| Anovulation or dysovulation (%) | 0 (0) | 2 (3.9) | 0 (0) | 0 (0) | 2 (1.0) |
| Number of attempts |  |  |  |  |  |
| 1 and 2 (%) | 35 (70.0) | 48 (94.1) | 41 (83.7) | 51 (100) | 175 (87.1) |
| 3 and 4 (%) | 15 (30.0) | 3 (5.9) | 8 (16.3) | 0 (0) | 26 (12.9) |
| IVF (%) | 20 (40.0) | 31 (60.8) | 28 (57.1) | 26 (51.0) | 105 (52.2) |
| ICSI-VF (%) | 30 (60.0) | 20 (39.2) | 21 (42.9) | 25 (49.0) | 96 (47.8) |
