# Supplementary figures and images for: Apolipoprotein B is regulated by gonadotropins and constitutes a predictive biomarker of IVF outcomes
Source: Reprod Biol Endocrinol. 2016 May 21;14:28. doi: 10.1186/s12958-016-0150-4 (PMC4875704; doi:10.1186/s12958-016-0150-4)

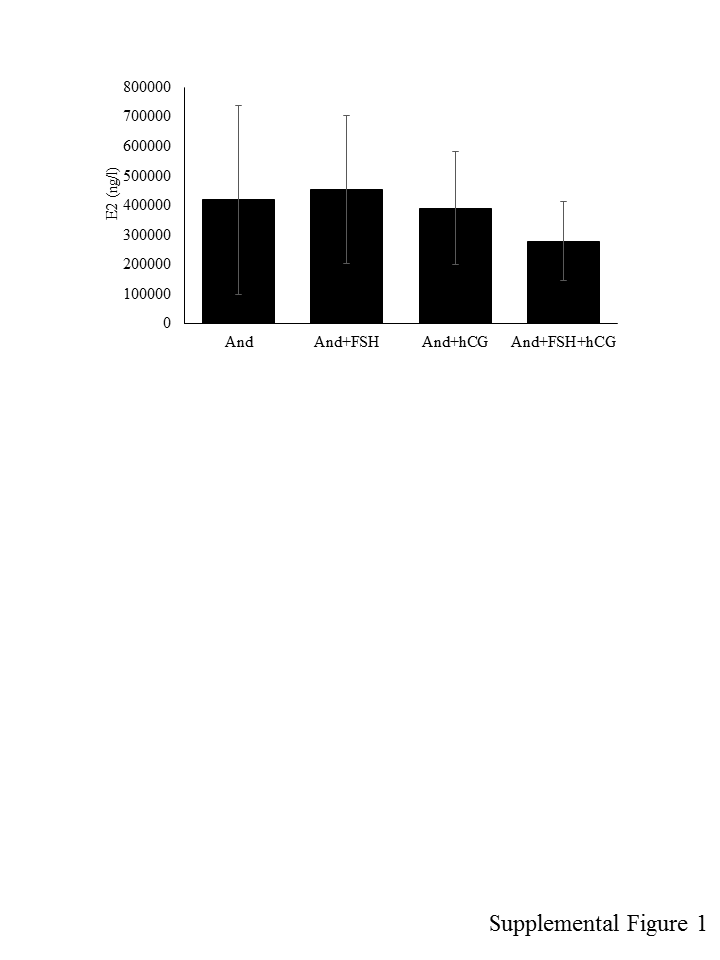

Supplement: Additional file 3: Figure S1. — 17 β-estradiol quantification (ng/l) into culture medium by immuno-chemiluminescence. Androstenedione (And) was added to the culture medium to provide specific substrate to granulosa cells, for oestrogen synthesis. (TIF 69 kb) [file 12958_2016_150_MOESM3_ESM.tif]

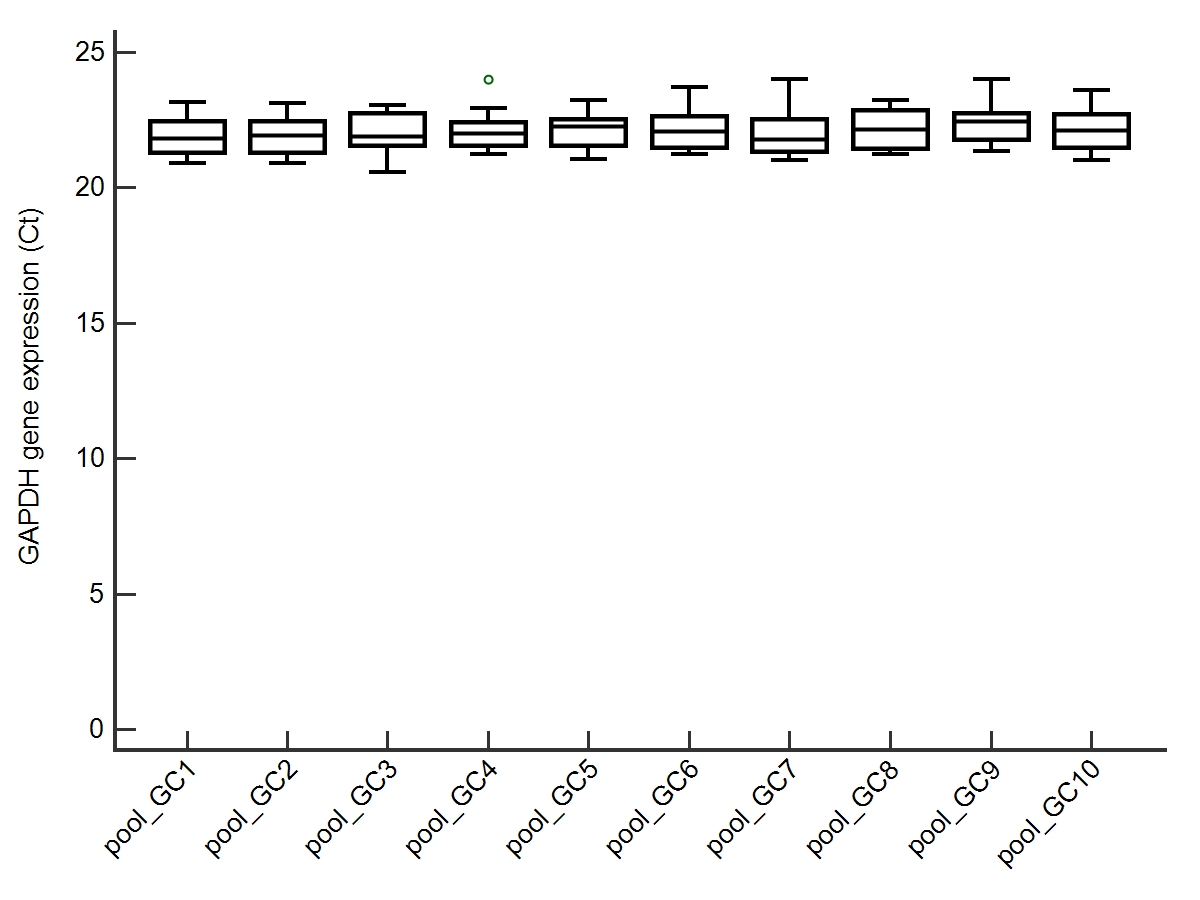

Supplement: Additional file 4: Figure S2. — The constant GAPDH gene expression in GC pools (n = 10) under our experiment conditions. All Ct-values were reported for the different GC pools from FF samples. (JPG 137 kb) [file 12958_2016_150_MOESM4_ESM.jpg]
